# Cardiac Repolarization and Autonomic Regulation during Short-Term Cold Exposure in Hypertensive Men: An Experimental Study

**DOI:** 10.1371/journal.pone.0099973

**Published:** 2014-07-01

**Authors:** Heidi Hintsala, Tuomas V. Kenttä, Mikko Tulppo, Antti Kiviniemi, Heikki V. Huikuri, Matti Mäntysaari, Sirkka Keinänen-Kiukaannemi, Risto Bloigu, Karl-Heinz Herzig, Riitta Antikainen, Hannu Rintamäki, Jouni J. K. Jaakkola, Tiina M. Ikäheimo

**Affiliations:** 1 Center for Environmental and Respiratory Health Research, University of Oulu, Oulu, Finland; 2 Medical Research Center Oulu, Oulu University Hospital and University of Oulu, Oulu, Finland; 3 Institute of Clinical Medicine, Department of Internal Medicine, University of Oulu, Oulu, Finland; 4 Department of Exercise and Medical Physiology, Verve Research, Oulu, Finland; 5 Institute of Health Sciences, University of Oulu, Oulu, Finland; 6 Unit of General Practice, Oulu University Hospital, Oulu, Finland; 7 Medical Informatics and Statistics Research Group, University of Oulu, Oulu, Finland; 8 Institute of Biomedicine, Department of Physiology and Biocenter of Oulu, University of Oulu, Oulu, Finland; 9 Department of Psychiatry, Kuopio University Hospital, Kuopio, Finland; 10 Oulu City Hospital, Oulu, Finland; 11 Finnish Institute of Occupational Health, Oulu, Finland; 12 Department of Medicine, Oulu University Hospital, Oulu, Finland; 13 Department of Applied Science, London South Bank University, London, England; Johns Hopkins University SOM, United States of America

## Abstract

**Objectives:**

The aim of our study was to assess the effect of short-term cold exposure, typical in subarctic climate, on cardiac electrical function among untreated middle-aged hypertensive men.

**Methods:**

We conducted a population-based recruitment of 51 hypertensive men and a control group of 32 men without hypertension (age 55–65 years) who underwent whole-body cold exposure (15 min exposure to temperature −10°C, wind 3 m/s, winter clothes). Conduction times and amplitudes, vectorcardiography, arrhythmias, and heart rate variability (autonomic nervous function) were assessed.

**Results:**

Short-term cold exposure increased T-peak to T-end interval from 67 to 72 ms (p<0.001) and 71 to 75 ms (p<0.001) and T-wave amplitude from 0.12 to 0.14 mV (p<0.001) and from 0.17 to 0.21 mV (p<0.001), while QTc interval was shortened from 408 to 398 ms (p<0.001) and from 410 to 401 ms (p<0.001) among hypertensive men and controls, respectively. Cold exposure increased both low (from 390 to 630 ms^2^ (p<0.001) and 380 to 700 ms^2^ (p<0.001), respectively) and high frequency heart rate variability (from 90 to 190 ms^2^ (p<0.001) and 150 to 300 ms^2^ (p<0.001), respectively), while low-to-high frequency-ratio was reduced. In addition, the frequency of ventricular ectopic beats increased slightly during cold exposure. The cold induced changes were similar between untreated hypertensive men and controls.

**Conclusions:**

Short-term cold exposure with moderate facial and mild whole body cooling resulted in prolongation of T-peak to T-end interval and higher T-wave amplitude while QTc interval was shortened. These changes of ventricular repolarization may have resulted from altered cardiac autonomic regulation and were unaffected by untreated hypertension.

**Trial Registration:**

ClinicalTrials.gov NCT02007031

## Introduction

Wintertime is associated with increased morbidity and mortality and a majority of this is related to cardiovascular causes [1], [2], such as myocardial infarctions [3], ruptures/dissection of aortic aneurysms [4], heart failures [5], as well as strokes [6]. Also ventricular arrhythmias [7] and sudden cardiac death [8] exhibit seasonal changes among cardiac patients, with higher occurrence during cold season.

Whole body exposure to cold and the resulting superficial cooling activates the sympathetic nervous system [9] that increases heart rate (HR) and constricts peripheral vasculature reducing heat loss from human to the environment. Sympathetic activation in general may produce altered cardiac function involving a higher risk of arrhythmias and cardiac events [10]. Facial cooling, on the other hand, is known to increase the vagal tone through the trigeminal nerve stimulation with a decrease in HR [11]. This may serve as a protective cardiovascular effect. However, for instance during whole body cold water immersion [12] or facial cooling [13] a co-activation of sympathetic and parasympathetic nervous system is observed. This causes conflicting inotropic and chronotropic drives to the heart (autonomic conflict) which may have an additional arrhythmogenic effect over the sympathetic activation alone [12], [13].

At present, the effects of short-term cold exposure with only superficial cooling on cardiac electrical function are not well known. Previous studies using pronounced whole-body cooling and hypothermia have detected electrocardiographic (ECG) manifestations such as the J (Osborne) waves, interval prolongation, T-wave abnormalities, and atrial and ventricular arrhythmias [12], [14], [15]. Furthermore, exercise in a cold environment induces pronounced cardiac symptoms in patients with cardiac disease [16], [17] and impairs post exercise cardiovagal regulation among healthy subjects [18].

Cold-induced arrhythmias might be pronounced in patients with hypertension in which sympathetic nervous system is already overactive [19]. Hypertension itself is a risk factor for arrhythmias, such as atrial fibrillation [20]. In addition, both short [21], [22] and long term [23] exposure to cold elevate blood pressure (BP) mainly as a result of the increased sympathetic activity. Hence, this augmented sympathetic drive could further aggravate the course of hypertension and increase the risk for arrhythmias and associated adverse health events. However, no previous studies explored the effect of cold exposure to cardiac electrical function among hypertensive subjects.

The objective of the present study was to assess the effects of short-term cold exposure similar to everyday winter circumstances on cardiac electrical function, incident arrhythmias, and the autonomic nervous system among subjects with untreated hypertension. We hypothesized that, compared to a warm environment, a cold exposure would increase sympathetic drive and induce electrophysiological changes known to be associated with arrhythmias. We also expected these changes to be pronounced among subjects with hypertension. The primary outcome measure of the study was ECG and vectorcardiographic features and secondary measure arrhythmias. The information of how cold exposure affects cardiac electrical function is relevant for medical professionals involved with treating cardiovascular diseases.

## Materials and Methods

### Subjects

**Figure 1:**
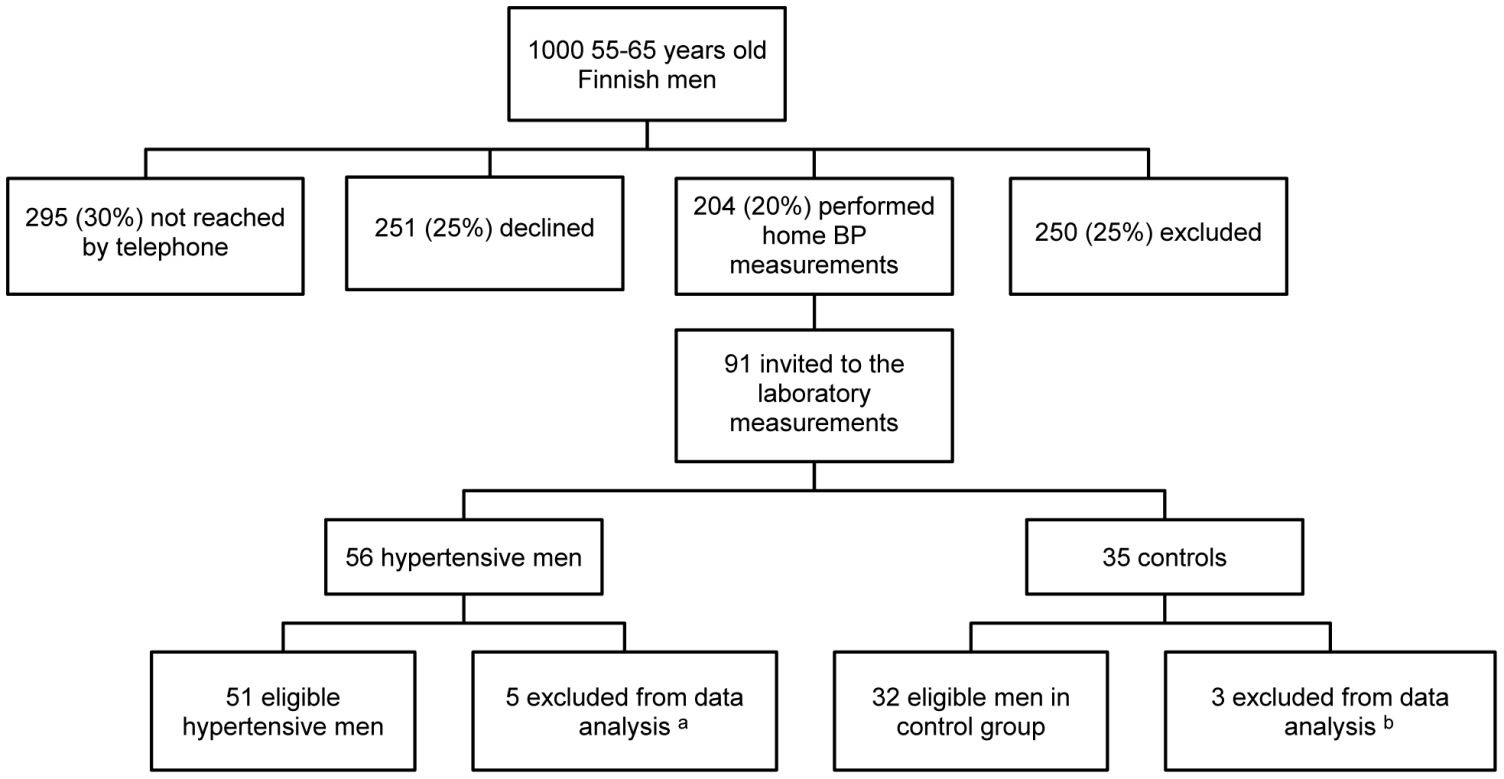
The recruitment procedure. BP, blood pressure. ^a^ Four subjects excluded for the usage of BP medication and one for inadequate data quality.^b^ One subject excluded for the usage of BP medication and two did not perform cold exposure for safety reasons.

We conducted a population-based recruitment (Figure 1) from April to November in 2011 which has been previously described in detail [21]. Briefly, men aged 55–65 years were randomly chosen from the population register in Oulu, Finland (65°N, 25°E), interviewed for eligibility and performed home BP measurements according to the recommendations of the European Society of Hypertension [24]. According to the sample size estimation and power analysis (G-Power 3.1.0) we estimated to detect statistically significant differences in brachial BP between a warm and cold environment [Power (1-β err prob), 0.9, Cohen's effect size 0.8, α err prob 0.05] in 23 participants. We aimed at a 2∶1 ratio of hypertensive men and control subjects for possible subgroup analyses among hypertensive, i.e. 46 hypertensive and 23 men in control group in final data-analyses. Based on the home BP measurements 83 subjects were enrolled into the study: 51 hypertensive men (the mean of 28 systolic BP measurements ≥135 and/or the mean of 28 diastolic measurements BP ≥85 mmHg) and 32 controls (the mean of 28 systolic/diastolic BP measurements <135/85 mmHg) (Table 1). The exclusion criteria of the study were presence of coronary heart disease or respiratory disease, use of antihypertensive drugs for any indication, an average home BP ≥175/105 mm Hg, no home BP measurements, inadequate data quality in the laboratory measurements (n = 1), and having a respiratory infection a week before exposure measurements. The other clinical characteristics did not differ among the groups except of 4 subjects had type 2 diabetes in the hypertensive group, and the hypertensive men had higher weight, body-mass index (BMI), and fat percentage. The study was approved by the ethics committee of Oulu University Hospital and all participants of the study gave written informed consent. The protocol for this trial and supporting Trend checklist are available as supporting information; see Checklist S1, Protocol S1, and Protocol S2. The trial was not registered prospectively because this observational controlled study does not any involve any clinical intervention. The authors confirm that all ongoing and related trials for this intervention are registered.

**Table 1:** Characteristics of the study group.

Variable	Hypertensive, N = 51	Controls, N = 32	P-values
Age, years	60±3	60±3	p = 0.90
Height, cm	177±6	176±6	p = 0.89
Weight, kg	84±9	79±11	p = 0.02
BMI, kg/m^2^	27±3	25±3	p = 0.01
BF, %	25±6	21±6	p = 0.01
SBP, mmHg	143±9	120±8	-
DBP, mmHg	86±6	73±6	-
Sokolow-Lyon index, mV	1.8±0.6	1.9±0.6	p = 0.51
Estimated VO2max, ml/kg/min	37±6	38±7	p = 0.19
Diabetes mellitus, n	4	0	-
Ever smoker, n	28	20	p = 0.56
Alcohol consumption ≥1 time/month, n	35	25	p = 0.42

Continuous variables are presented as mean values ± standard deviation, categorical variables as number of cases. BMI, body mass index; BF, body fat percentage; SBP, systolic blood pressure in home measurements; DBP, diastolic blood pressure in home measurements; estimated VO2max, indirectly estimated maximal oxygen uptake; and Sokolow-Lyon index, estimate of left ventricular hypertrophy.

### Experimental Protocol

The experimental measurements were performed from August to November 2011 before the start of the cold season by trained professionals. The experiments began with a short introduction of the measurement protocol and visit to the climatic chamber. Height was measured. Body composition was assessed by bioelectrical impedance analysis (InBody 720 Biospace Ltd, Korea) and physical fitness was measured while resting with the Polar Own Index Fit Test (Polar S610, Polar, Finland). After the measurements the study subjects were equipped with thermistors (skin temperature), ECG electrodes, and a cuff for measuring brachial BP and dressed with winter clothing (ca. 2 clo during cold exposure and 1.6 clo during baseline and recovery measurements [25]). The exposure protocol consisted of three consecutive 15 minutes phases: baseline, cold exposure, and recovery period in baseline conditions. The subjects were exposed to cold in a wind tunnel (air temperature *T_a_* = −10°C, air velocity *V_a_* = 3 m/s, and relative air humidity *H_a_* = 50%) adjacent to a climatic chamber in which baseline and recovery measurements were performed (*T_a_* = 18°C, *V_a_*<0.2 m/s, *H_a_* = 30%). All measurements were performed during the office hours.

### Digital ECG Recordings

Derived 12-lead ECG recordings (Medilog AR12, Huntleigh Healthcare, Austria) were performed in order to measure the electrical function of the heart throughout the experiment. Recordings were made using 16 bit amplitude resolution and 256 Hz sampling rate.

### ECG and Vectorcardiographic Features

The signal processing and analysis were carried out with custom-made software written in Matlab (MathWorks, inc., Natick, MA, USA). Baseline wander was removed with cubic spline interpolation and high-frequency noise was suppressed with a bidirectional 40 Hz low-pass filter. Ectopic and abnormally shaped beats were removed from the analysis. Beats were considered ectopic if the preceding RR interval differed by more than 20% from the last valid RR interval. Beats with deviating morphology were identified with template matching. The template was updated with each accepted beat and the threshold for acceptance was 95% match. Subsequently, the signal was resampled from 256 Hz to 512 Hz in order to improve the morphological alignment of the beats. All beats were aligned in each of the leads based on cross-correlation (time alignment between the leads was not changed). The aligned beats were then filtered with a ten-beat wide sliding window in order to produce representative median beats for each cardiac cycle and to suppress the noise within the beats.

Three representative median beats were taken five minutes apart from each other, starting from the beginning of each phase, i.e. baseline, cold and recovery phases. In order to assure the accuracy of the interval measurements, these samples were analyzed manually by a trained operator, who identified the P-wave onset, QRS boundaries, T-wave peak and T-wave offset from each of the 12 leads for each sample. Subsequently, PR, QRS, QT and T-peak to T-end intervals were calculated in leads II and V5. QT interval was corrected for HR with nomogram method (QTc) [26]. Left ventricular hypertrophy (LVH) was evaluated by Sokolow-Lyon voltage criteria. LVH was defined as a sum of S amplitude in lead V1 and R amplitude in lead V5 or V6 (lead with higher amplitude was chosen) ≥3.5 mV.

Mean spatial QRS-T angle and maximum T-wave amplitude were calculated in a beat-to-beat manner. For this purpose, the QRS boundaries, T-wave peak and T-wave offset were located automatically for each beat. The QRS onset and offset detection was based on the method described by Daskalov and Cristov [27], utilizing the morphology of the ECG in the determination of the QRS boundaries. T-wave offset was defined as the intersection of the isoelectric line and a line fitted by least squares to the maximum slope of the T-wave. Isoelectric line was defined as the mean amplitude of the preceding and following TP intervals. The maximum amplitude between QRS offset and T-wave offset was taken as the T-wave peak (minimum T-wave amplitude if inverted T-wave was observed).

Mean QRS and T axes were calculated from the orthogonal X, Y and Z leads, which were reconstructed from the 12-lead ECG samples with inverse-Dower matrix [28]. The mean spatial axes were based on the position vectors defined by the areas of the QRS and T-wave loops in the X, Y and Z leads and the mean spatial QRS-T angle was defined as the angle between these axes. Additionally, the maximum T-wave amplitude was calculated from leads V5 and II. However, instead of lead V5, lead V4 or V6 (which ever had the highest amplitude of T-wave) was used if the amplitude in V5 was below 0.15 mV.

### Arrhythmias

We also analyzed the frequency of single ventricular and atrial ectopic beats. Automatic detection of ectopic beats was performed from baseline, cold exposure, and recovery ECG recordings with Medilog Darwin Holter Analyses software version V1.13.4 (TOM Medical Handels Gmbh, Austria). Automatic detection of ectopic beats was based on Medilog ADAPT algorithm [29]. Prematurity threshold was set to 15% compared to three normal preceding beats. Detected ectopic beats were confirmed with visual inspection by trained health care professionals.

### Heart Rate Variability

Heart rate variability (HRV) analyses were performed in time and frequency domain as described previously [30] (Hearts 8, Heart Signal Co., Oulu, Finland) to assess activity of the autonomic nervous system. Artifacts and ectopic beats were removed and replaced with the local average. Linear de-trending was applied in measured RR interval data. Fast Fourier transform was applied to estimate the power spectral densities of the RR interval variability. Low (LF, 0.04–0.15 Hz) and high frequency power (HF, 0.15–0.40 Hz) of RR interval oscillations were quantified. The ratio between LF and HF powers (LF/HF-ratio) was also analyzed. The power spectral components of HRV were computed in 5 min periods and then averaged over each 15 min phase, i.e. baseline, cold, and recovery. To analyze the overall HRV, we computed the standard deviation of all normal RR intervals (SDNN) in time domain for each 15 minutes phase.

### Blood Pressure

Brachial BP was measured with the use of oscillometric sphygmomanometry (Schiller BP 200 Plus, Schiller, Switzerland) at 3-minute intervals during baseline, cold exposure, and recovery measurements. The arm of the subject was supported and the position of the cuff was at the level of the heart.

### Skin Temperatures and Thermal Sensations

Skin temperature was measured continuously with the use of thermistors (NTC DC95, Digi-Key, Thief River Falls, MN, USA) and data was recorded at 12 s intervals with an eight channel temperature data logger (SmartReaderPlus, Acr Systems Inc., BC, Canada). The thermistors were placed on the middle finger, back of the hand, shoulder blade, and cheek. Thermal perception for the whole body and face was assessed using subjective judgement scales [31].

### Statistical Methods

Subject characteristics between study groups were compared with independent t–test for continuous variables and chi-square test for categorical variables. Sensitivity analyses were conducted separately for BMI, weight, fat percentage and chronic diseases. Due to lack of effect to the results these were ignored in further analyses. Means between baseline, cold, and recovery as well as study groups were compared by 2-way repeated measures ANOVA and contrast tests. Parameters with non-Gaussian distribution were transformed into natural logarithm. In addition, we executed linear regression analyses to evaluate the rate-dependency (HR changes) of T-wave amplitude change. The results are expressed as means and their standard deviations (SD) or 95% confidence intervals (CI). Statistical analyses were performed with IBM SPSS for Windows version 19 (IBM Corp. Released 2010, Armonk, NY, USA) and significance was set at p<0.05.

## Results

### Thermoregulatory Responses

The employed cold exposure involved facial cooling which could be demonstrated as a rapid (30°C to 21°C in 2 min) and robust (30°C to 15°C in 10 min) reduction in skin temperature of the cheek. In general, the winter clothing slowed down or even prevented superficial cooling of most areas of the body, as demonstrated by the only slightly lowered shoulder blade temperature (baseline 34°C vs. cold 31°C). Finger temperatures decreased by 3–4°C in the cold (26°C to 22°C). Skin temperatures on cheek and finger remained 4–7°C lower 10–15 minutes after the exposure compared to baseline measurements both in hypertensive men and controls. Skin temperature responses during baseline, cold exposure and the recovery measurements did not differ between hypertensive men and control group. Thermal perceptions (median) of the whole body and face were neutral during baseline and recovery measurements and cool during cold exposure in both study groups.

### Cold exposure, ECG, HRV, BP, and arrhythmias

**Figure 2:**
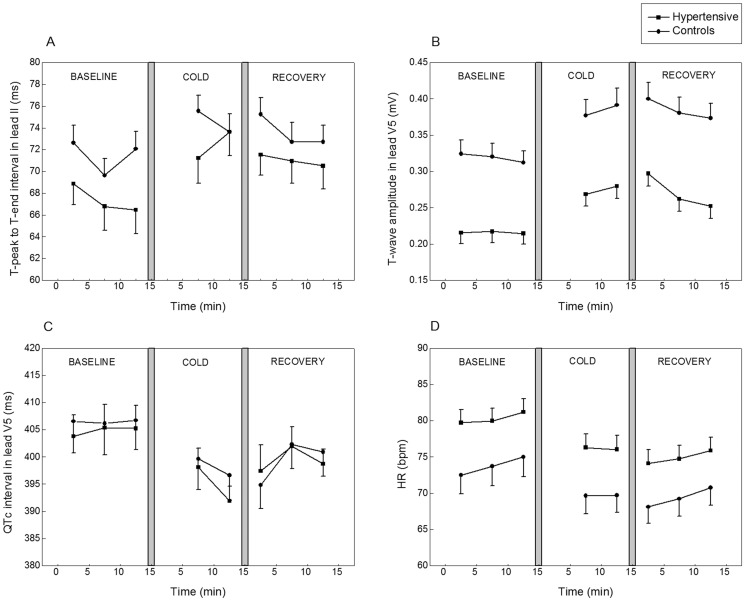
ECG changes during cold exposure. T-peak to T-end interval in lead II (A), T-wave amplitude in lead V5 (B), QTc in lead V5 (C), and HR (D) of hypertensive men (N = 51) and controls (N = 32) before (baseline), during, and after (recovery) cold exposure. Values represent means and standard error of the means.

**Figure 3:**
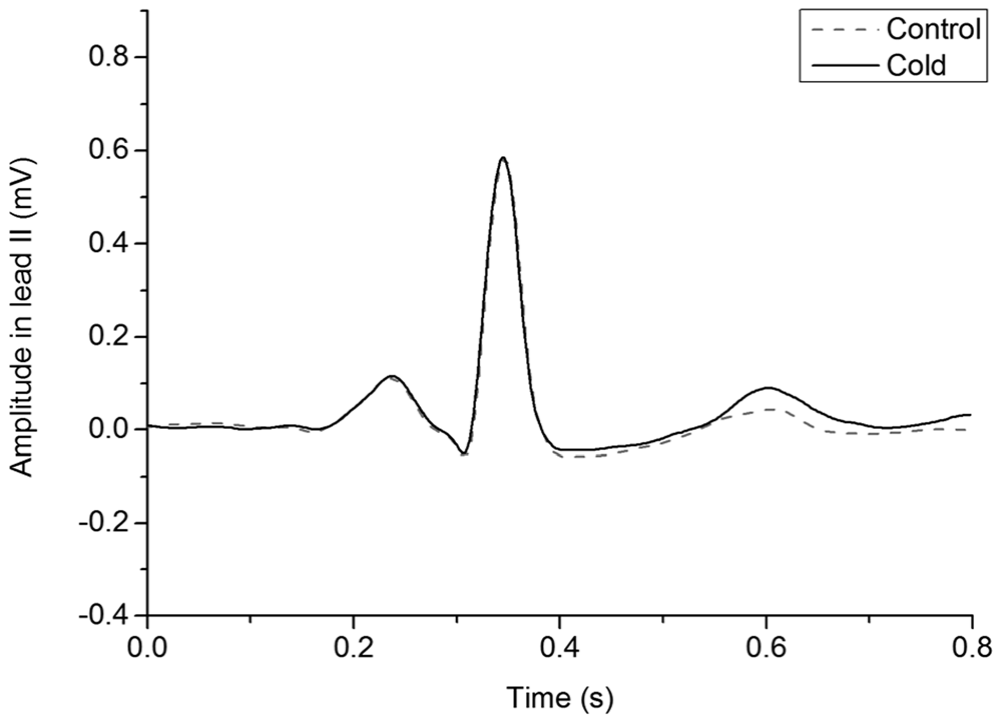
Example ECG presenting changes in cardiac repolarization. Figure presents a median ECG from 10 consecutive beats as an example of changes in T-wave in lead II before (baseline), during, and after (recovery) cold exposure. Cold exposure increased T-peak to T-end interval and T-wave amplitude.

The detected changes in T-wave amplitude, T-peak to T-end interval, QTc, and HR are depicted in Figure 2. Cold exposure resulted in higher maximum T-wave amplitude compared to baseline in both hypertensive and control subjects in leads II and V5 (Table 2, Figure 2). We also examined the HR dependency of this parameter and estimated the following regression equation: T-wave amplitude change = 48.370+0.426 * RR change (adjusted R^2^ = 0.296, p<0.001). QRS-T angle and QTc interval decreased during cold exposure in both groups (Table 2). T-peak to T-end interval increased in lead II (Figure 2), but remained constant in lead V5. An example ECG presenting changes in cardiac repolarization during cold exposure is depicted in Figure 3. PR and QRS intervals were mainly unaltered. Cold exposure increased systolic (26–27 mmHg) and diastolic BP (12 mmHg) both among hypertensive and control subjects. Cold induced ECG changes did not return to the baseline level during 15 minutes recovery follow-up after the exposure (Table 2). Cold exposure increased the frequency of ventricular, but not atrial ectopic beats (Table 2). In all phases, frequency of ectopic beats was low in both study groups.

**Table 2:** Cardiovascular responses to cold exposure among hypertensive men and control group.

Variable	Hypertensive, N = 51				Controls, N = 32			
	Baseline (Mean±SD)	Cold Exposure (Mean±SD)	Recovery (Mean±SD)	Effect of cold exposure (Mean, 95% CI)	Baseline (Mean±SD)	Cold Exposure (Mean±SD)	Recovery (Mean±SD)	Effect of cold exposure (Mean, 95% CI)
HR, bpm†	80±13	77±14*	75±13*	−3 (−5 to −2)	73±15	70±14*	69±13*	−4 (−6 to −1)
T-peak (II), mV†	0.12±0.06	0.14±0.06*	0.14±0.07*	0.03 (0.02 to 0.03)	0.17±0.06	0.21±0.07*	0.21±0.07*	0.04 (0.02 to 0.05)
TpTe (II), ms	67±14	72±16*	71±13*	5 (3 to 7)	71±8	75±8*	74±8*	3 (0 to 6)
QT (II), ms†	368±27	366±30	369±31	−1 (−4 to 2)	381±31	382±30	383±29	1 (−4 to 6)
QTc (II), ms	408±27	398±19*	402±24*	−10 (−16 to −5)	410±31	401±28*	403±24*	−10 (−13 to −6)
PR (II), ms	153±17	154±20	153±19	2 (0 to 3)	155±22	156±20	155±23	2 (−3 to 7)
QRS (II), ms†	97±12	96±13*	97±13	0 (−2 to 1)	92±9	90±9*	91±9	1 (−1 to 2)
T-peak (V5), mV†	0.22±0.11	0.27±0.12*	0.27±0.12*	0.06 (0.05 to 0.07)	0.32±0.10	0.38±0.13*	0.38±0.12*	0.07 (0.04 to 0.09)
TpTe (V5), ms	80±15	81±12	82±13	1 (−2 to 3)	80±13	80±9	80±9	0 (−4 to 3)
QT (V5), ms†	365±27	364±31	367±30	−1 (−4 to 2)	377±31	379±29	379±29	2 (−3 to 7)
QTc (V5), ms	405±27	395±20*	399±23*	−10 (−16 to −4)	406±32	398±28*	400±25*	−8 (−12 to −5)
PR (V5), ms	152±17	154±17	153±18	2 (0 to 3)	154±22	156±21	153±24	2 (−3 to 7)
QRS (V5), ms	90±13	90±14	91±13	0 (−2 to 1)	89±10	90±10	89±10	1 (−1 to 2)
QRS-T angle, °	56±48	48±47*	48±46*	−7 (−2 to −12)	41±48	37±46*	39±47*	−3 (−6 to −1)
VEB, count/15 min	1.3±4.0	1.8±4.2*	1.4±3.9	0.5 (0.0 to 1.0)	0.4±0.9	0.7±1.6*	0.3±0.9	0.3 (−0.2 to 0.7)
AEB, count/15 min	0.6±2.7	0.7±3.9	0.5±2.7	0.2 (−0.4 to 0.7)	0.1±0.4	0.1±0.2	0.1±0.3	−0.1 (−0.2 to 0.1)
VEB2, count/15 min	2.3±5.2	3.2±5.3*	2.5±5.1	1.0 (0.0 to 1.9)	0.9±1.3	1.6±2.1*	0.8±1.3	0.7 (−0.5 to 1.9)
AEB2, count/15 min	1.0±3.7	1.3±5.2	0.9±3.6	0.3 (−0.7 to 1.3)	0.3±0.6	0.2±0.4	0.2±0.4	−0.2 (−0.6 to 0.3)
Systolic BP, mmHg†	148±13	175±16*	153±13*	27 (24 to 30)	126±13	152±14*	133±12*	26 (21 to 30)
Diastolic BP, mmHg†	92±10	104±10*	94±9*	12 (10 to 14)	79±10	91±10*	83±8*	12 (10 to 14)
LF RR, ms^2^	390±420	630±600*	550±480*	240 (120 to 360)	380±290	700±650*	580±500*	320 (140 to 500)
HF RR, ms^2^	90±110	190±220*	150±160*	100 (60 to 150)	150±200	300±370*	220±220*	150 (50 to 250)
LF/HF-ratio†	6.4±4.8	4.6±2.9*	5.2±4.2*	−1.9 (−3.0 to −0.7)	4.3±3.2	3.4±1.8*	4.0±3.2*	−0.9 (−2.0 to 0.2)
SDNN RR, ms^2^	32±12	40±16*	39±16*	8 (6 to 11)	33±12	41±8*	43±18*	8 (3 to 13)

Values are group means over each phase (baseline, cold, and recovery) ± standard deviations (SD). Effect of cold exposure: mean of intra-individual differences between “Cold exposure” and “Baseline” with 95% confidence intervals (95% CI). HR, heart rate; T-peak, T-wave amplitude; TpTe, T-peak to T-end interval; QT, QT-interval; QTc, QT adjusted to HR; PR, and QRS, corresponding intervals on ECG; QRS-T angle, spatial angle between QRS and T; VEB, ventricular and AEB, atrial ectopic beats; VEB2, ventricular and AEB2, atrial ectopic beats for those who had ectopic beats during the measurements (n = 28 hypertensive and 13 controls); BP, blood pressure; LF, low frequency; HF, high frequency; LF/HF, the ratio of LF and HF; SDNN, standard deviation of all normal RR intervals; .

* p<0.05 vs. baseline (time),

† p>0.05 vs. hypertensive (group).

There were no significant difference in responses to cold between hypertensive men and controls (no time×group effect).

When examining the HRV parameters, cold exposure increased both LF (60–80%) and HF (100–110%) components of HRV, as well as SDNN (25%) in both hypertensive and control subjects (Table 2). LF/HF-ratio and HR decreased during cold exposure in both groups. HRV parameters did not return to the baseline level during 15 minutes recovery follow-up after cold exposure.

### Hypertension, ECG, HRV, BP, and arrhythmias

There were no differences in ECG parameters or arrhythmias between the study groups, except for T-wave amplitude which was ca. 0.1 mV higher in controls compared with hypertensive men during all phases (p<0.001) and for unadjusted QT (p<0.05). HR was lower (6–7 beats) and LF/HF-ratio higher among hypertensive compared to control subjects. Otherwise, there were no differences in the HRV parameters between the study groups. Hypertensive subject had a higher SBP and DBP compared to controls during all phases (p<0.001), as expected.

### Cold, hypertension and changes in ECG, HRV, BP, and arrhythmias

All cold induced changes were comparable among hypertensive men and controls (no time×group effect).

## Discussion

The present study is the first to assess cardiac electrical activity, cardiovascular autonomic regulation, and arrhythmias in hypertensive middle-aged men during cold exposure similar to everyday winter circumstances in a subarctic climate. We observed that cold exposure slightly increased T-peak to T-end interval, T-wave amplitude and decreased QTc interval reflecting changes in ventricular repolarization. Simultaneously an autonomic co-activation with increased sympathetic and parasympathetic activity during the cold exposure was observed. The frequency of ventricular ectopic beats increased slightly in the cold. These cold-related responses were similar in hypertensive and control subjects.

According to our results, short-term cold exposure not involving substantial whole body cooling increased T-peak to T-end interval in lead II, reflecting prolongation of cardiac repolarization. The observed increase in T-wave amplitude, on the other hand, may indicate dispersion in duration of action potentials [32]. In addition, cold exposure had no effect on QT interval despite a reduction in HR. The frequency of ventricular ectopic beats was slightly increased during cold exposure, which may reflect the observed cardiac electrical changes.

Previous experimental or clinical studies examining the effects of cold on cardiac electrical function have involved intense whole-body exposures, such as hypothermia [14], [15], [33] and immersion to cold water [12] or employed exercise in cold [16]–[18]. These studies have shown several different effects on ECG, such as atrial [12], [14], [15], [33] and ventricular [15], [33] arrhythmias, interval prolongation [14], [15], [33], T-wave abnormalities [14], [15], Osborne waves [14], [15], [33], and pronounced ST-depression during exercise among cardiac patients [16], depending on cold exposure duration and its intensity. Only a few studies have examined cardiac electrical activity under superficial or local cold exposure, such as facial cooling with [34] or without breath holding [13].

The observed changes in our study in cardiac electrophysiology during cold exposure are based on current knowledge caused by an altered autonomic nervous system activity [9], [12], [13], [18], [35]. This altered autonomic regulation persisted throughout the 15 minutes recovery period. One plausible reason for this could be lowered skin temperatures at recovery, which could have provided a continuing parasympathetic and sympathetic stimulus. Also, irrespective of skin temperature, the autonomic nervous system regulation may not recover immediately after the cessation of stressor. The applied cold exposure increased sympathetic activity [9], as demonstrated by the increased peripheral BP and cardiac work [21]. Furthermore, the spectral analysis of HRV showed that both LF and HF increased while HR decreased as a sign of parasympathetic activation during cold exposure. Sympathetic stimulation increases T-peak to T-end interval in limb leads [36]. Sympathetic stimulation results in either inverted or positive T-wave depending on the stimulated area [37], [38]. Sympathetic activation also increases risk of arrhythmias [10]. The observed shortened cold-related QTc interval with reduced HR but unaltered QT interval in the present study probably reflects the simultaneous effects of increased vagal and sympathetic activity [35], [39].

Sympathovagal co-activation of autonomic nervous system is a regulatory process but could cause conflicting pressures on the heart and may partially explain the observed increases in T-peak to T-end interval, T-wave amplitude, and ventricular arrhythmias. Such co-activation has been previously described in association with stimuli, like facial cooling [13], during cold water immersion involving breath holding [12], as well as during the recovery phase of exercise [40]. The co-activation of the sympathetic and vagal outflow changes HR dynamics from more fractal to random HR organization [13] and could predispose to arrhythmias [12], [41].

Hypertension is related to increased sympathetic activity [19], [42] and is a risk factor of cardiac events such as arrhythmia [20] and ischemic changes [20], [43]. In our study, untreated hypertensive men had higher HR and LF/HF- ratio and lower T-wave amplitude compared to controls in both warm and cold conditions, consistent with previous non-cold related studies [44], [45] and indicating elevated sympathetic activity in hypertension. Sympathetic hyperactivity combined with the cold stressor could potentate adverse ECG changes during cold exposure. Contrary to our hypothesis, we did not observe major differences in cardiac responses to cold exposure between hypertensive men and controls. The lack of major ECG changes may be due to a short disease history among the untreated hypertensive subjects in our study. It is possible that in persons whose cardiovascular disease is at a more advanced stage, disturbed circulatory adjustments or compromised cardiac oxygen supply could lead to aggravated cardiovascular responses and impaired cardiac function.

ECG parameters are commonly rate-dependent [46], and some of the observed changes in our study could be explained by slightly decreased HR in the cold. According to the linear regression model less than one third of the observed change in T-wave amplitude could be explained by change in HR in our study, i.e. most of the change is related to other factors. T-peak to T-end interval has been presented to have minor or no connection with HR [46]. In contrast, QT interval is known to be strongly dependent of HR [46] and rate dependency of it varies with altered autonomic function [35], [39]. QRS-T angle decreased with decreasing HR in our study, consistent with a previous study assessing rate-dependency of QRS-T-angle during exercise test [47].

### Strengths and limitations

The strength of our study is that all subjects were drawn from the general population and thus the results reflect typical cardiovascular response to cold among untreated middle aged hypertensive men and men without hypertension. Furthermore, we were able to produce a strictly controlled and equal cold exposure to all subjects. Hence, we consider that our results have public health implications due to the population-based sample and the utilized cold exposure which was similar to everyday winter circumstances in a cold climate.

Performing a randomized controlled trial would have reduced possible bias related to anticipation caused by measurements. However, we emphasized familiarization and reduction of stress of all participants caused by the experimental conditions. Another limitation of our study was that some of the study participants had other chronic diseases in addition to hypertension. However, we conducted sensitivity analyses by excluding the participants with these chronic diseases, but did not detect any effects on the main results. Also, despite the population based recruitment, some selection may still occur through non-participation. We do not have information of the characteristics of the subjects declining to participate in the study.

### Conclusions

In conclusion, our results demonstrate that moderate short-term whole body cold exposure commonly occurring in everyday life [48] results in altered cardiac repolarization among middle-aged men. Rather than being of clinical importance, the detected changes may be physiological responses to short-term cold exposure. Furthermore, as compared to the control group, the untreated hypertensive men are not more susceptible to cold induced changes in cardiac electrical function. However, the detected cold effects could be more substantial with more intense cold exposure or among patients with cardiac disease. Future studies are warranted to examine cardiac electrical function during cold exposure among persons with advanced cardiovascular diseases.

## Supporting Information

Checklist S1 TREND statement checklist.
(DOC)

Protocol S1 The study protocol approved by ethics committee – original version in Finnish.
(DOC)

Protocol S2 The study protocol approved by ethics committee – English version.
(DOC)
