# Supplementary material for: Cardiac Repolarization and Autonomic Regulation during Short-Term Cold Exposure in Hypertensive Men: An Experimental Study
Source: PLoS One. 2014 Jul 1;9(7):e99973. doi: 10.1371/journal.pone.0099973 (PMC4077657; doi:10.1371/journal.pone.0099973)
Supplement: Protocol S1 — The study protocol approved by ethics committee – original version in Finnish. (DOC) [file pone.0099973.s002.doc]

# Protocol S2. The study protocol approved by ethics committee – original version in Finnish

**TUTKIMUSHANKKEEN YHTEENVETO, EETTINEN TOIMIKUNTA**

**TUTKIMUKSEN TAUSTA JA TARKOITUS**

Verenpainetauti on yksi merkittävimmistä kansanterveydellisistä haasteistamme. Suomalaisista aikuisista noin puolella on kohonnut verenpaine (Antikainen ym. 2006). Kohonnut verenpaine lisää riskiä sydän- ja verisuonitautitapahtumille.

Kylmä kohottaa verenpainetta kaikilla ihmisillä välittömästi sille altistuttaessa. Tämän lisäksi verenpaine on pysyvästi koholla kylmänä vuodenaikana (Brennan ym. 1982, Mitchell ym. 2002, Modesti ym. 2006, Sun 2010). Maailmanlaajuiset tutkimukset ovat osoittaneet verenpaineen olevan käänteisesti verrannollinen lämpötilaan ja vaihtelevan voimakkaasti vuodenajan mukaan (Alpérovitch ym. 2009, Barnett ym. 2005). Kohonnut verenpaine on keskeinen kylmästä aiheutuvan sairastavuuden ja kuolleisuuden riskitekijä. Kuitenkaan tällä hetkellä ei hyvin tunneta millaisia verenpainetautia sairastavien henkilöiden verenpainevasteet ovat kylmässä. Kokeelliset tutkimukset ovat osoittaneet, että henkilöillä joilla verenpaine on lievästi koholla, voidaan lääkityksen avulla paremmin ylläpitää suositusarvojen mukaisia verenpainetasoja myös kylmässä (Komulainen 2007).

**TUTKIMUKSEN TAVOITTEET**

Tutkimuksen kiinnostuksen kohteina on kohonnutta verenpainetta sairastavat henkilöt (ei vielä hoitoa) ja hankkeessa selvitetään eroaako heidän verenpainevasteensa terveiden henkilöiden vasteista kylmäaltistustilanteessa, joka toteutuu jokapäiväisessä elämässä.

Yksilöidyt tavoitteet:

1. tutkia kuinka paljon ja kuinka nopeasti verenpaine kohoaa kylmässä sekä onko tämä vaste erilainen kohonnutta verenpainetta sairastavilla verrattuna terveisiin henkilöihin.
2. selvittää kauanko verenpaineen palautuminen kestää altistuksen jälkeen.
3. selvittää kuinka eri kehon osien kylmäaltistus vaikuttaa verenpainevasteisiin.
4. selvittää mitkä tekijät ovat yhteydessä verenpainemuutoksiin (esim. veren hyytymistekijät).

**TUTKIMUKSEN MERKITYS**

Tutkimus tarjoaa uutta tietoa verenpainevasteista kylmässä kohonnutta verenpainetta sairastavilla. Tutkimustiedon avulla voidaan tarjota terveydenhuollon henkilöstölle työkaluja, kuinka ohjeistaa kohonnutta verenpainetta sairastavia henkilöitä tarkoituksenmukaiseen toimintaan kylmässä. Lisäksi tutkimuksessa saadaan uutta tietoa kylmän vaikutuksista verenpaineen dynamiikkaan kohonnutta verenpainetta sairastavilla, mikä auttaa heidän oikean lääkityksen kohdentamisessa. Tällainen kylmän terveyshaittojen hallintamalli voi vähentää tai ehkäistä kohonnutta verenpainetautia sairastavien kylmästä aiheutuvia terveyshaittoja, kuten valtimosairauksiin liittyviä oireiluja: sydänkohtauksia, aivoverenvuotoja tai -infarkteja.

**TUTKIMUSAINEISTO JA MENETELMÄT**

**AINEISTO**

Tutkimusaineisto koostuu Oulun seudun 55–65-vuotiaista hoitamattomista kohonnutta verenpainetta sairastavista sekä terveistä miehistä.

**Rekrytointi**

Aineisto seulotaan Oulun kaupungin väestöstä, jolloin tutkittavat poimitaan väestörekisteristä satunnaisotannalla. Otantaan sisällytetään 55–65-vuotiaita miehiä. Tutkittaviin otetaan yhteyttä puhelimitse ja selvitetään lyhyellä haastattelulla, onko heillä lääkärin toteama kohonnut verenpaine, sepelvaltimotauti tai hengitystiesairauksia (kuten astma tai keuhkoahtaumatauti). Näitä henkilöitä ei sisällytetä tutkimukseen, koska halutaan tutkia kohonnutta verenpainetta, joka on hoitamatta sekä sulkea pois tutkimuksesta riskiryhmiin kuuluvat henkilöt. Miehiltä, jotka eivät ilmoita kohonnutta verenpainetta kysytään lupaa tulla mittaamaan verenpaine. Tutkittavan perusverenpainetaso määritetään kotimittauksella niin, että tutkittava itse mittaa verenpaineensa aamulla ja illalla kahdesti viikon ajan (Käypä Hoito –ohje). Mittausten keskiarvo kertoo verenpainetason. Jos kotimittauksessa todetaan kohonnut verenpaine (≥ 140/90 mmHg), kysytään henkilön halukkuutta osallistua tutkimukseen. Kotimittauksen perusteella tutkittavat jaetaan potilasryhmään (korkea verenpaine, mutta ei lääkitystä) ja verrokkiryhmään (normaali verenpaine).

Jos tutkittavalla todetaan kotimittauksessa korkea normaali verenpaine (≥ 130/85 mmHg), annetaan hänelle suositus ottaa yhteyttä terveydenhuoltoon. Tällöin sairauden selvittäminen ja hoito siirtyvät hoidosta vastaavalle lääkärille. Tutkittavaa kannustetaan samanaikaisesti osallistumaan tutkimukseen. Jos tutkittavan kotimittauksessa todetaan kriittisesti korkea verenpaine (≥ 180/110 mmHg), ohjataan hänet ensihoitoon ja jätetään pois tutkimuksesta.

**Otoskoko**

Otoskoon laskemisessa oletettiin, että verenpaineen hajonta 55–65-vuotiailla miehillä Oulun seudulla on 21/11 mmHg (Finriski 2002 perusraportti) ja verenpaine nousee -5⁰C kylmäaltistuksen aikana keskimäärin 20/20 mmHg (Komulainen 2007). Tavoitellaan 90 % voimaa ja tyypin I virhetasoksi valittiin 0.05. Funktion f(a,b) arvoksi saatiin näin 10.5 ja otoskooksi ((2 x (21 mmHg)^2^) / (20 mmHg)^2^) x 10.5 = 23 tutkittavaa ryhmää kohden. Ryhmiin valitaan vähintään 30 tutkittavaa riittävän aineiston turvaamiseksi aineiston keruun päätyttyä. Tutkimukseen halutaan kohonnutta verenpainetta sairastavia suhteessa terveisiin 2:1, joten potilasryhmän otoskooksi saadaan 60. Yhteensä tutkittavia halutaan rekrytoida näin ollen 90.

Kohonnutta verenpainetta sairastavia on 25–64-vuotiaista suomalaisista miehistä noin 50 % ja heistä hoitamattomia on noin 1/3 (Kastarinen ym. 2009). Näin ollen hoitamaton kohonnut verenpaine on noin 17 % aikuisista miehistä. Väestörekisteristä seulottavien määrä on 0.17 x N = 60 → N = 353 tutkittavaa. Koska seulonnassa ja tutkimuksen aikana jää pois tutkittavia, seulotaan 500 miestä väestörekisteristä.

**MENETELMÄT**

**Kysely**

Tutkittavien taustatiedot selvitetään kyselylomakkeella heidän suostuttuaan osallistumaan tutkimukseen. Selvitetään laajasti verenpaineeseen vaikuttavia tekijöitä: ammatti, terveydentila, tupakointi, alkoholin käyttö, ruokavalio, liikunta-aktiivisuus, mieliala ja kylmätuntemukset (ks. Kyselylomake).

**Kylmäaltistus**

Ennen kylmäaltistusta tutkittavat valmistellaan: iholämpötila-anturit, verinäyte, keskeisen verenkierron paine, valtimojäykkyys ja verenpaine lämpimässä. Stressin vaikutus verenpaineeseen pyritään minimoimaan niin, että tutkittava saa tutustua kammioon ennen mittausta. Koko kehon 15 minuutin kylmäaltistus kontrolloiduissa olosuhteissa lämpöolosuhdekammiossa, jossa ilman lämpötila on -5⁰C ja tuulen nopeus 2 m/s, talvivaatetus. Tutkittava seisoo paikallaan altistuksen ajan. Altistuksen päätyttyä tutkittavilta mitataan uudestaan valmisteluvaiheen muuttujat.

**Mitattavat muuttujat**

1. Verenpaine (ambulatorinen mittaus ennen, jälkeen ja kylmäaltistuksen aikana)
2. Keskeisen verenkierron paine ja valtimojäykkyys (pulse wave velocity)
3. Iholämpötilat: 6 eri kehon pisteestä (raajat, kädet, vartalo)
4. Verinäyte ennen kylmäaltistusta. Näytteestä määritetään terveyteen liittyviä taustamuuttujia kuten pieni verenkuva, hyytymätekijät ja rasva-arvot. Mahdollisesti tehdään myös geneettisiä määrityksiä, jotka tutkisivat geeniperimän vaikutusta kylmän ja kohonneen verenpaineen yhteyteen.
5. Lämpötuntemukset, -viihtyvyys sekä kylmäoireet (kylmäaltistuksen aikana)
6. Kehonkoostumus (InBody) bioimpedanssimittauksella
7. Aerobinen kunto: Levossa toteutettava kuntotesti (Polar Own Index)

**TUTKIMUKSEN TOTEUTUS JA AIKATAULU**

Tutkimusaineiston rekrytointi ja pilottimittaukset toteutetaan keväällä 2011. Varsinaiset mittaukset toteutetaan elo-syyskuussa 2011. Aineisto analysoidaan ja raportoidaan vuosien 2012 ja 2013 aikana.

**LÄHTEET**

Alpérovitch ym. Arch Intern Med 2009;169:75-80.

Antikainen ym. Eur J Cardiovascular Prev Rehab 2006;13:13-29.

Barnett ym. J Epidemiol Community Health 2005;59:551-7.

Brennan ym. BMJ 1982;285:919-23.

Kastarinen ym. J Hypertens. 2009 Aug;27(8):1552-9.

Komulainen. Acta Universitatis Ouluensis. 2007 D949 Medica. ISBN 978-951-42-8612-4.

Mitchell ym. Int J Epidemiol 2002;31:831-8.

Modesti ym. Hypertenstion 2006;47:155-161.

Sun ym. Fron Biosci 2010;1:495-503.
