# Supplementary material for: Cardiac Repolarization and Autonomic Regulation during Short-Term Cold Exposure in Hypertensive Men: An Experimental Study
Source: PLoS One. 2014 Jul 1;9(7):e99973. doi: 10.1371/journal.pone.0099973 (PMC4077657; doi:10.1371/journal.pone.0099973)
Supplement: Protocol S2 — The study protocol approved by ethics committee – English version. (DOC) [file pone.0099973.s003.doc]

**Protocol S3. The study protocol approved by ethics committee – English version**

**SUMMARY OF THE STUDY PROTOCOL, ETHICAL COMMITTEE**

**BACKGROUND OF THE STUDY**

Hypertension is one of the most important national health concerns and approximately half of the middle-aged adults in Finland have high blood pressure (Antikainen et al. 2006). Hypertension increases the risk for cardiovascular events.

Cold temperature causes an immediate rise in blood pressure among all people. In addition, blood pressure remains at a higher level during the cold season (Brennan et al. 1982, Mitchell et al. 2002, Modesti et al. 2006, Sun 2010). Global studies have shown an inverse relation between temperature, as well as high seasonal variation in blood pressure (Alpérovitch et al. 2009, Barnett et al. 2005). An elevated blood pressure is an important determinant of the cold-related morbidity and mortality. However, blood pressure responses to cold exposure among hypertensive subjects are not yet well-known. Experimental studies have shown that blood pressure medication results in lower blood pressure level during cold exposure among subjects with mild hypertension (Komulainen 2007).

**AIM OF THE STUDY**

The aim of the study is to assess blood pressure responses among (untreated) hypertensive people during cold exposure similar to everyday winter circumstances. Responses will be compared to blood pressure changes among healthy individuals.

The specific goals are:

1. to assess the magnitude and timing of blood pressure increase during cold exposure and to compare the responses between hypertensive and healthy subjects.
2. to assess the timing of blood pressure recovery after the cold exposure.
3. to assess the effect of cooling of different parts of the body to blood pressure.
4. to assess the factors affecting blood pressure changes (e.g. blood coagulation factors).

**SIGNIFICANCE OF THE STUDY**

The study provides novel knowledge on blood pressure dynamics during cold exposure among hypertensive people. This knowledge is useful for healthcare professionals for advising hypertensive patients for proper cold protection or for medical doctors considering medication for their hypertensive patients. The knowledge provided by the study can be applied to reduce or prevent cold-related health events among hypertensive patients, such as myocardial infarctions and strokes.

**MATERIAL AND METHODS**

**MATERIAL**

The study subjects are men with untreated high blood pressure or healthy men. They are 55-65 years old residents of the city of Oulu, Finland.

**Recruitment**

A random sample is drawn from the Finnish Population Register Centre from residents of Oulu (55-65 years old men). Subjects are contacted by phone and a short interview is applied to evaluate the eligibility of the subjects. People with diagnosed/treated high blood pressure, coronary artery disease (CAD) or respiratory disease are excluded because the study aims for examining persons with untreated hypertension and excluding other cold-related health risk factors. Eligible men are asked for consent to perform blood pressure measurements. Subjects measure their blood pressure at home according to national guidelines: two measurements in the morning and evening daily for one week. According to the mean value of those 28 measurements, the subjects are classified either to patient (≥ 140/90 mmHg, high untreated blood pressure) or control (≥ 130/85 mmHg, normal blood pressure) group.

The subjects with high normal blood pressure in home measurements (≥ 130/85 mmHg) are advised to contact their health care center or health care professionals, who will carry out the diagnosis and initiate treatment for high blood pressure. These subjects are simultaneously encouraged to attend to the experimental study. The subjects with blood pressure ≥ 180/110 mmHg are advised to contact emergency care and they are excluded from the study.

**Sample size**

Assumptions for sample size estimation: Variation of blood pressure among the study subjects (men, aged 55-65 years, residents of Oulu) is 21/11 mmHg (FINRISK 2002-basic report). An average increase in blood pressure during -5⁰C cold exposure is 20/20 mmHg (Komulainen 2007).

90 % power was aimed and 0.05 was chosen for type I error bound. This resulted to function f (a,b) value of 10.5 and sample size ((2 x (21 mmHg)^2^) / (20 mmHg)^2^) x 10.5 = 23 subjects / group. Each group should consist of minimum of 30 subjects to have sufficient amount of data for the analyses. A 2:1 ratio of hypertensive and normotensive subjects is aimed, which results into patient group of 60 subjects and total amount of 90 subjects.

The prevalence of hypertension is ca. 50% among 25-64 years old men in Finland and ca. 1/3 of them are untreated (Kastarinen et al. 2009), i.e. prevalence of untreated hypertension is ca. 17% among adult Finnish men. Sample size for screening of the subjects from the Finnish Population Register Centre is 0.17 x N = 60 → N = 353 subjects. To consider the loss of follow-up, a sample of 500 men will be applied.

**METHODS**

**Questionnaire**

The study subjects fill in a questionnaire to assess for factors possibly affecting blood pressure, such as occupation, health status, smoking, alcohol consumption, diet, physical activity, mental condition, and cold sensation (questionnaire attached).

**Cold exposure**

The study subjects are prepared with skin temperature sensors and a blood sample is drawn before cold exposure. Also, central blood pressure, arterial stiffness and blood pressure in warm environment are measured. Measurement excitement is minimized by introduction and habituation to the experimental conditions before the measurements. During the experiment a 15 minutes whole body cold exposure with winter clothing is implemented in controlled circumstances in a climatic chamber with air temperature of -15ºC and wind of 2m/s. The subjects stand still during the exposure. The subjects are followed in warm conditions after the cold exposure.

**Parameters to be measured**

1. Blood pressure (ambulatory measurements before, during, and after cold exposure)
2. Central blood pressure and arterial stiffness (pulse wave velocity)
3. Skin temperature: 6 measurement points (limbs, body)
4. Blood sample before cold exposure. Health related parameters will be analyzed from blood sample, such as red and white blood cells, coagulation factors, and blood lipids. We might perform genetic analyses to evaluate relation of cold temperature and high blood pressure.
5. Thermal sensations and comfort, cold related symptoms (during cold exposure)
6. Body composition with bio-impedance measurement (InBody)
7. Aerobic fitness: physical fitness measurements while resting (Polar Own Index)

**IMPLEMENTATION AND SCHEDULE OF THE STUDY**

The recruitment of the study subjects and pilot measurements are performed during spring 2011. The experimental measurements are implemented from August to September 2011. Collected data will be analyzed in 2012 and 2013.

**RECERENCES**

Alpérovitch et al. Arch Intern Med 2009;169:75-80.

Antikainen et al. Eur J Cardiovascular Prev Rehab 2006;13:13-29.

Barnett et al. J Epidemiol Community Health 2005;59:551-7.

Brennan et al. BMJ 1982;285:919-23.

Kastarinen et al. J Hypertens. 2009 Aug;27(8):1552-9.

Komulainen. Acta Universitatis Ouluensis. 2007 D949 Medica. ISBN 978-951-42-8612-4.

Mitchell et al. Int J Epidemiol 2002;31:831-8.

Modesti et al. Hypertenstion 2006;47:155-161.

Sun et al. Fron Biosci 2010;1:495-503.
